# Risk factors of pulmonary embolism in postpartum women

**DOI:** 10.3389/fmed.2025.1617052

**Published:** 2025-10-06

**Authors:** Min Cui, Yan Wu, Shanshan Tong, Qiang Feng, Xuewei Qin, Xue-ling Wu

**Affiliations:** ^1^Department of Pharmacy, Renji Hospital, School of Medicine, Shanghai Jiao Tong University, Shanghai, China; ^2^Department of Pharmacy, Taicang TCM Hospital Affiliated to Nanjing University of Chinese Medicine, Jiangsu, China; ^3^Department of Pharmacy, Tongji Hospital, Tongji University, Shanghai, China; ^4^Department of Pharmacy, Xiamen Fifth Hospital, Fujian, China; ^5^Department of Obstetrics, Obstetrics and Gynecology Hospital, School of Medicine, Tongji University, Shanghai, China; ^6^Department of Respiratory Medicine, Renji Hospital, School of Medicine, Shanghai Jiao Tong University, Shanghai, China

**Keywords:** pulmonary embolism, postpartum woman, risk factors, retrospective study, d-dimer level

## Abstract

**Introduction:**

Pulmonary embolism (PE) is a potentially fatal complication, especially in postpartum women.

**Objective:**

To explore the risk factors of PE in postpartum women.

**Methods:**

A retrospective cohort study consisted of 130 postpartum women with PE (PE Group) and 192 postpartum women without PE (non-PE Group), which were analyzed.

**Results:**

The PE group was older, with 37.7% having multiple deliveries, and the inter-pregnancy interval was mostly 1–3 years (*p* < 0.05). The incidence of prophylactic anticoagulation, anemia, prenatal fever, emergency cesarean section, twins, postpartum acute infection, intraoperative bleeding, postpartum hemorrhage, and venous thromboembolism (VTE) was significantly higher in the PE group. Abnormal clinical manifestations and echocardiographic measures were more common (*p* < 0.05). The levels of creatinine, urea nitrogen, fibrinogen, total protein, and albumin in the PE group were significantly lower, whereas total bilirubin, d-dimer, and B-type Natriuretic Peptide (BNP) were significantly higher (*p* < 0.05). The ROC curve analysis showed the best cutoff point for d-dimer level within postpartum women was 2.24 mg/L (specificity 90.1%, sensitivity 88.9%). On multivariate analysis, VTE and abnormal echocardiography were independent risk factors for PE (*p* < 0.05). The d-dimer levels (OR 1.363, 95% CI 1.716–8.907, *p* = 0.001) were a specific marker for clinical monitoring of PE.

**Conclusion:**

It was necessary to strengthen the clinical monitoring of PE-related risk factors in postpartum women, especially in those with VTE, abnormal echocardiography, and a d-dimer level > 2.24 mg/L.

## Introduction

Pulmonary embolism (PE) is one of the main causes of death during pregnancy (1). Perinatal women are four to five times more likely to develop PE than their non-perinatal peers (2). In recent years, with the rapid development of our country’s economy, the relaxation of the three-child policy, and the increase in assisted reproduction, the incidence of PE has also increased. PE was a major cause of maternal death in China, especially in postpartum women, when the risk of fatal PE was higher (1).

However, PE is difficult to diagnose in the clinical setting, because even in the absence of PE, pregnant women often experience breathing difficulties, chest pain, and tachycardia (3). D-dimer is the first choice for pulmonary embolism diagnosis, but the threshold for routine d-dimer is not suitable for perinatal women and lacks specificity. Furthermore, computer tomography pulmonary angiography (CTPA) should be avoided in pregnancy, which may cause potential harm to the pregnant women and fetus (4, 5).

In addition, the risk factors of PE in postpartum women are rarely available to date. Therefore, a retrospective case–control study was conducted on postpartum women diagnosed with PE in this study. Clinical data were collected and analyzed to identify the related risk factors for PE, and a multivariate logistic regression analysis was performed. This was done to enhance the clinical monitoring of postpartum women and prevent the occurrence of PE.

## Methods

### Inclusive criteria

A total of 130 postpartum women diagnosed with PE were admitted to Shanghai Renji Hospital and Shanghai First Maternity and Infant Hospital between July 2020 and November 2022 and enrolled in this retrospective case–control study. They were distributed in a case group (PE group). In the same period, 192 postpartum women who were delivered in the above medical institutions and did not develop PE were selected as the control group (non-PE group).

Inclusive criteria are as follows: (1) According to the “Chinese expert consensus on the treatment of acute pulmonary embolism by multidisciplinary team” and “Guidelines for the diagnosis, treatment and prevention of pulmonary thromboembolism,” (4, 6) postpartum women with pulmonary embolism were diagnosed. (2) All postpartum women in the groups were examined by echocardiography. (3) Postpartum women with suspected risk factors for PE, such as with suspicious symptoms of PE or with multiple high-risk factors, should be investigated with CTPA. (4) The informed consent of the postpartum woman or her family was obtained for the examination and treatment. (5) All of the relevant data were intact.

Postpartum women with vague diagnoses, incomplete clinical data, and a previous diagnosis of PE were excluded from this study. Non-thrombotic embolism, such as amniotic fluid embolism, air embolism, and fat embolism, was also excluded.

### Data acquisition

Those following clinical parameters were collected from postpartum women: basic information, such as age, height, weight, body mass index (BMI), systolic blood pressure (SBP), diastolic blood pressure (DBP), and neonatal weight; Reproductive history, such as *in vitro* fertilization (IVF), number of pregnancies, number of births, and interval between births; Thrombus-related diseases, such as previous deep venous thrombosis, antiphospholipid syndrome, high risk of hereditary thrombosis, family history of thrombotic disease, and varicose veins; Previous illnesses, such as hypertension, diabetes, cancer, connective tissue disease, and cardiovascular disease; Pregnancy complications; Delivery characteristics.

The relevant laboratory indices at diagnosis, including platelet count, creatinine, urea nitrogen, estimated glomerular filtration rate (eGFR), alanine transaminase (ALT), aspartate transaminase (AST), total bilirubin (TB), direct bilirubin (DB), fibrinogen, d-dimer, total protein, albumin, and BNP.

The prophylactic anticoagulation, clinical features, imaging data, anticoagulation therapy, and laboratory indexes after treatment of the postpartum women were collected.

### Statistical analysis

All statistical analysis was conducted using SPSS software, version 26.0 (SPSS Inc., Chicago, IL, USA). The Kolmogorov–Smirnov test was used to assess the normality of data. Descriptive data were expressed as medians with interquartile range (IQR), and continuous variables were compared using the Mann–Whitney test. Categorical variables were presented by counts (n) and percentages, and count data were presented using the χ^2^ test with Yates’s correction or Fisher’s exact test as necessary. In all analyses, we preliminarily confirmed the effect of multicollinearity of the covariates used in the statistical analysis. Univariate logistic regression analysis was used to investigate variables potentially associated with the occurrence of PE. All variables with statistical significance in the univariate analysis and variables clinically related to the occurrence of PE were included in the multivariate logistic regression analysis, and stepwise regression analysis (forward: LR method) was performed; *p* < 0.05 (two-sided) was considered statistically significant. Multivariable stepwise logistic regression analysis was adopted to further identify the independent risk factors for postpartum women combined with PE. The associations between d-dimer and PE were expressed as ROC curve analysis.

## Results

### Basic information

The basic information is listed in Table 1. This study included 130 postpartum women with confirmed PE and 192 postpartum women without PE. Both groups met the inclusive criteria. The median age of postpartum women in the case group was 33.0 years (30.0–35.0), and the average age of the control group was 31.0 years (28.3–34.0) (*p* = 0.004). There was no significant difference between the two groups in weight, height, BMI, SBP, DBP, newborn weight, IVF, or number of pregnancies (*p* > 0.05). Most of the women in the non-PE group had not given birth before this pregnancy (76.0% vs. 62.3%, *p* = 0.008). 37.7% of the women in the PE group had at least one child before the current pregnancy (p = 0.008). Among them, 25 (19.2%, *p* < 0.05) women had an interbirth interval of 1–3 years. There was no significant difference between the two groups in the previous illness (*p* > 0.05), such as thrombosis-related diseases and medical complications.

**Table 1:** Basic information about both groups of postpartum women.

Basic information	non-PE Group (*n* = 192)	PE Group (*n* = 130)	*z*/*x*^2^	*p*
Age / (years)	31.0 (28.3, 34.0)	33.0 (30.0, 35.0)	−2.918	0.004
Weight (childbirth) / (kg)	67.5 (62.6, 74.8)	71.3 (64.0, 76.0)	−1.299	0.194
Height (childbirth) / (m)	1.6 (1.6, 1.7)	1.6 (1.6, 1.7)	−0.250	0.802
BMI (childbirth) / (kg/m^2^)	25.9 (24.1, 29.3)	27.0 (24.9, 29.6)	−1.561	0.118
BMI^a^				
Normal / (%)	116.0 (60.4)	66.0 (50.8)	2.936	0.087
Overweight / (%)	50.0 (26.0)	42.0 (32.3)	1.491	0.222
Fat / (%)	6.0 (3.1)	4.0 (3.1)	0.000	1.000
Weight (before pregnancy) / (kg)	56.0 (50.5, 62.0)	57.5 (52.0, 63.8)	−0.612	0.541
Height (before pregnancy) / (m)	1.6 (1.6, 1.7)	1.6 (1.6, 1.7)	−0.011	0.991
BMI (before pregnancy) / (kg/m^2^)	21.2 (19.9, 23.6)	22.5 (19.9, 24.6)	−0.938	0.348
SBP / (mmHg)	120.0 (112.0, 125.0)	118.0 (110.0, 126.0)	−0.951	0.342
DBP / (mmHg)	75.0 (70.0, 80.0)	75.0 (69.0, 82.0)	−0.456	0.648
Newborn weight / (g)	3215.0 (2860.0, 3520.0)	3172.5 (2562.5, 3500.0)	−1.098	0.272
IVF / (%)	24.0 (12.5)	21.0 (16.2)	0.861	0.354
Number of pregnancies				
1 time / (%)	80.0 (41.7)	57.0 (43.8)	0.151	0.698
> 1 time / (%)	112.0 (58.3)	73.0 (56.2)	0.151	0.698
Number of births				
0 time / (%)	146.0 (76.0)	81.0 (62.3)	7.030	0.008
≥ 1 time / (%)	46.0 (24.0)	49.0 (37.7)	7.030	0.008
Interval between births				
N0: first child / (%)^b^	146.0 (76.0)	81.0 (62.3)	7.030	0.008
1–3 years / (%)	3.0 (1.6)	25.0 (19.2)	30.478	<0.05
4–5 years/ (%)	11.0 (5.7)	5.0 (3.8)	0.582	0.446
6–10 years / (%)	23.0 (12.0)	10.0 (7.7)	1.549	0.213
> 11 years / (%)	9.0 (4.7)	9.0 (6.9)	0.734	0.392
Previous illness				
Thrombosis-related diseases / (%)^c^	2.0 (1.0)	5.0 (3.8)	1.700	0.192
Medical complications / (%)^d^	53.0 (27.6)	29.0 (22.3)	1.146	0.284

a Normal BMI: < 28 kg/m^2^, Overweight: 28–34.9 kg/m^2^, Fat: ≥ 35 kg/m^2^.

b N0: first child.

c Thrombosis-related diseases: Deep venous thrombosis (DVT), Antiphospholipid syndrome, Genetic predisposition to high risk of thrombosis, Family history of thrombotic disease, Varicose veins.

d Medical complications: High blood pressure, Diabetes, Cancer, Connective tissue disease, Cardiovascular disease, Kidney disease, Inflammatory bowel disease, Obesity, hepatitis, Anemia, Liver dysfunction, Hypothyroidism, Malignancy, Hashimoto’s thyroiditis, Autoimmune disease.

### Clinical features

There was no significant difference in the incidence of elevated d-dimer during pregnancy between the two groups (*p* > 0.05), but 57.4% of the women in the PE group received prophylactic anticoagulation (*n* = 74.0, *p* < 0.05) (Table 2). The incidence of anemia and prenatal fever in the PE group was higher than that in the non-PE group (*p* < 0.05). There was no significant difference in pregnancy complications between the two groups (*p* > 0.05), such as preeclampsia, eclampsia, pregnancy-induced hypertension, gestational diabetes, overweight during pregnancy, hypothyroidism, rheumatism-related diseases, placental chorioangioma, abnormal liver function, proteinuria, ovarian cyst, postpartum fever, thrombophilia, fatty liver, genital wart, and combined medication.

**Table 2:** Clinical features of both groups of postpartum women.

Clinical features	non-PE Group (*n* = 192)	PE Group (*n* = 130)	*z*/*x*^2^	*p*
Pregnancy complications^a^				
No pregnancy complications / (%)	103.0 (53.6)	72.0 (55.4)	0.094	0.759
Preeclampsia/Eclampsia / (%)	10.0 (5.2)	13.0 (10.0)	2.683	0.101
Pregnancy-induced hypertension / (%)	11.0 (5.7)	10.0 (7.7)	0.490	0.484
Gestational diabetes / (%)	38.0 (19.8)	16.0 (12.3)	3.111	0.078
Overweight during pregnancy / (%)	7.0 (3.6)	4.0 (3.1)	0.000	1.000
D-dimer elevated during pregnancy / (%)	153 (79.7)	102 (78.5)	0.071	0.790
Prophylactic anticoagulation / (%)	72.0 (36.5)	74.0 (57.4)	13.633	<0.05
Hypothyroidism / (%)	16.0 (8.3)	6.0 (4.6)	1.683	0.194
Anemia / (%)	6.0 (3.1)	17.0 (13.1)	11.575	0.001
Rheumatism-related diseases / (%)	5.0 (2.6)	1.0 (0.8)	0.600	0.439
Placental chorioangioma / (%)	0.0 (0.0)	2.0 (1.5)	2.972	0.162
Abnormal liver function / (%)	4.0 (2.1)	3.0 (2.3)	0.000	1.000
Proteinuria / (%)	8.0 (4.2)	2.0 (1.5)	1.013	0.314
Ovarian cyst / (%)	2.0 (1.0)	2.0 (1.5)	0.000	1.000
Prenatal fever / (%)	0.0 (0.0)	8.0 (6.2)	9.710	0.002
Postpartum fever / (%)	0.0 (0.0)	2.0 (1.5)	2.972	0.162
Thrombophilia / (%)	0.0 (0.0)	1.0 (0.8)	1.482	0.404
Fatty liver / (%)	1.0 (0.5)	2.0 (1.5)	0.117	0.733
Genital wart / (%)	1.0 (0.5)	1.0 (0.8)	0.077	1.000
Merged medication history^b^				
Combined medication (pre-pregnancy) / (%)	13.0 (6.8)	3.0 (2.3)	3.270	0.071
Combined medication (post-pregnancy) / (%)	103.0 (53.6)	59.0 (45.4)	2.116	0.146
Characteristics and complications of delivery				
Mode of delivery				
Natural childbirth / (%)	87.0 (45.3)	27.0 (20.8)	20.417	<0.05
Midwifery / (%)	5.0 (2.6)	1.0 (0.8)	0.600	0.439
Emergency cesarean section / (%)	11.0 (5.7)	27.0 (20.8)	16.846	<0.05
Elective Caesarean section / (%)	88.0 (45.8)	73.0 (56.2)	3.303	0.069
Termination of pregnancy by intraamniotic injection / (%)	1.0 (0.5)	2.0 (1.5)	0.117	0.733
Normal gestational weeks / (%)^c^	162.0 (84.4)	96.0 (73.8)	5.396	0.020
Stillborn / (%)	3.0 (1.6)	2.0 (1.5)	0.000	1.000
Fetal intrauterine growth retardation / (%)	4.0 (2.1)	5.0 (3.8)	0.356	0.550
Twins / (%)	9.0 (4.7)	14.0 (10.8)	4.323	0.038
Deep vein catheterization / (%)	1.0 (0.5)	2.0 (1.5)	0.117	0.733
No activity > 3 d / (%)	1.0 (0.5)	5.0 (3.8)	3.045	0.081
Pre-eclampsia / (%)	9.0 (4.7)	8.0 (6.2)	0.333	0.564
Acute postpartum infection / (%)	2.0 (1.0)	9.0 (6.9)	6.442	0.011
Prenatal bleeding / (%)	5.0 (2.6)	7.0 (5.4)	0.985	0.321
Intraoperative hemorrhage / (%)	58.0 (30.2)	119.0 (91.5)	117.790	<0.05
Postpartum hemorrhage / (%)	5.0 (2.6)	20.0 (15.4)	17.681	<0.05
Plasma replacement / (%)	2.0 (1.0)	4.0 (3.1)	0.819	0.365
Use of antibiotics in the perinatal period / (%)^d^	84.0 (43.8)	66.0 (50.8)	1.535	0.215
VTE / (%)	1.0 (0.5)	89.0 (68.5)	177.677	<0.05
Clinical manifestations / (%)^e^	5.0 (2.6)	16.0 (12.3)	11.972	0.001
Hemoptysis / (%)	1.0 (0.5)	1.0 (0.8)	0.077	1.000
Chest distress / (%)	4.0 (2.1)	11.0 (8.5)	7.100	0.008
Chest pain / (%)	0.0 (0.0)	3.0 (2.3)	2.322	0.128
Shortness of breath / (%)	1.0 (0.5)	6.0 (4.6)	4.337	0.037
Abnormal echocardiography / (%)	10.0 (5.2)	55.0 (42.3)	66.220	<0.05

a No hyperemesis gravidarum in all parturients.

b Combined medication (pre-pregnancy): Metformin, Levothyroxine, Olanzapine, Cyclosporine, Risperidone, Aripiprazole, Hydroxychloroquine, Prednisone, Amlodipine, Atorvastatin, Aspirin, etc; Combined medication (post-pregnancy): Polyene phosphatidylcholine, Nifedipine, Aspirin, Ursodeoxycholic acid, Labetalol, Insulin, Prednisone, Hydroxychloroquine, Mercaptopurine, Levothyroxine sodium, Clopidogrel hydrogen sulfate, Metformin, etc.

c Weeks of gestation: Normal: 37–42 w, Preterm: < 37 w, Prolonged: > 42 w.

d Use of antibiotics in the perinatal period: Cefuroxime, Cefazolin, Ceftriaxone, Ceftazidime, Cefoxitin, Cefmetazole, Cefpirome, Levofloxacin, Meropenem, Morinidazole, etc.

e Clinical manifestations: Hemoptysis, Chest distress, Chest pain, Shortness of breath, Difficulty in breathing. No respiratory distress was observed in all the parturients. Some patients had ≥ 2 clinical manifestations.

In the non-PE group, most pregnant women delivered normally (45.3%, *n* = 87.0, *p* < 0.05) and had normal gestational age (84.4%, *n* = 162.0, *p* = 0.020). However, the incidence of emergency cesarean section was higher in the PE group (20.8%, *n* = 27.0, *p* < 0.05). The pregnancy rate of twins in the PE group was higher (10.8%, *n* = 14.0, *p* = 0.038), and acute postpartum infection (6.9%, *n* = 9.0, *p* = 0.011) was more common. There was no significant abnormality in the prepartum bleeding of the two groups of primiparas (*p* > 0.05). The incidence of intraoperative hemorrhage in the PE group (91.5%) was significantly higher than that in the non-PE group (30.2%, *p* < 0.05). The median blood loss in the PE group was 300 mL. The incidence of postpartum hemorrhage (15.7% vs. 2.6%, *p* < 0.05) in the PE group was also significantly higher than that in the non-PE group. However, the plasma replacement rate was similar in the two groups of parturients (1.0% vs. 3.1%, *p* = 0.365). There was no significant difference in the use rate of perioperative antibiotics in the two groups of parturients (*p* > 0.05).

More postpartum women in the PE group had VTE (68.5%, *n* = 89.0, *p* < 0.05), which was significantly different from the non-PE group. It was more common for women with clinical symptoms to be in the PE group (12.3% vs. 2.6%, *p* = 0.001). Most patients in the PE group had clinical manifestations of chest distress (8.5%, *p* = 0.008) and shortness of breath (4.6%, *p* = 0.037). Four women in the PE group exhibited two types of clinical symptoms, while one was in the non-PE group. CTPA showed that 25 (19.2%) postpartum women had PE in both lungs, 40 (30.8%) in the left lung, and 65 (50.0%) in the right lung. The pulmonary embolism severity index (PESI) was 33.4 (average). 42.3% (*n* = 55.0, *p* < 0.05) of the patients in the PE group had echocardiographic abnormalities.

According to the Guidelines for the diagnosis, treatment, and prevention of pulmonary thromboembolism (6), we stratified the risk of the patients. In this study, one patient had a high-risk condition, the other had an intermediate-risk condition, and the remaining patients had low-risk conditions.

### Laboratory indicators

The levels of creatinine (45.0 μmol/L), urea nitrogen (3.0 mmol/L), fibrinogen (4.0 mg/L), total protein (53.6 g/L), and albumin (30.3 g/L) in the PE group were significantly lower than those in the non-PE group (*p* < 0.05) (Table 3). The total bilirubin (8.8 μmol/L), d-dimer (5.0 μg/mL), and BNP (76.0 pg./mL) in the PE group were significantly higher than those in the non-PE group (p < 0.05). There were no significant differences in eGFR, ALT, AST, direct bilirubin, platelet count, cholesterol, and ferritin between the two groups (*p* > 0.05).

**Table 3:** The laboratory indices of postpartum women in the two groups at the time of diagnosis.

Laboratory indicators (at diagnosis)^a^	non-PE Group (*n* = 192)	PE Group (*n* = 130)	*z*/*x*^2^	*p*
Creatinine / (μmol/L)	47.0 (42.0, 54.0)	45.0 (40.0, 51.0)	−2.081	0.037
Urea nitrogen / (mmol/L)	3.6 (2.8, 4.3)	3.0 (2.4, 3.9)	−2.938	0.003
eGFR / (ml/(min × 1.73 m^2^))	105.0 (84.0, 121.0)	122.0 (120.0, 131.0)	−1.838	0.066
ALT / (U/L)	14.0 (9.0, 22.0)	13.0 (9.0, 19.5)	−0.946	0.344
AST / (U/L)	19.8 (15.0, 24.0)	21.0 (16.0, 26.0)	−1.696	0.090
Total bilirubin / (μmol/L)	5.8 (4.1, 7.3)	8.8 (6.8, 11.2)	−7.326	<0.05
Direct bilirubin / (μmol/L)	2.1 (1.6, 2.7)	1.9 (1.4, 2.6)	−1.576	0.115
Platelet count / (×10^9^/L)	203.5 (172.0, 241.0)	189.0 (158.0, 239.0)	−1.654	0.098
Fibrinogen / (mg/L)	4.3 (3.9, 4.7)	4.0 (3.4, 4.8)	−2.428	0.015
D-dimer / (μg/mL)	1.1(0.7,1.6)	5.0(3.7,10.5)	−13.287	<0.05
Cholesterol / (mmol/L)	5.8 (3.9, 6.9)	5.8 (5.0, 6.6)	−0.180	0.857
Total protein / (g/L)	58.9 (55.7, 62.3)	53.6 (50.5, 58.0)	−6.631	<0.05
Albumin / (g/L)	33.9 (32.1, 36.0)	30.3 (28.1, 32.8)	−7.610	<0.05
BNP / (pg/mL)	39.5 (25.6, 70.3)	76.0 (38.1, 162.3)	−3.080	0.002
Ferritin / (μg/L)	22.6 (13.0, 38.2)	25.1 (16.1, 40.1)	−0.536	0.592

a Creatinine 53–115 μmol/L, Urea nitrogen 2.5–6.5 mmol/L, ALT 0–40 U/L, AST 0–40 U/L, Total bilirubin 0–17.1 μmol/L, Direct bilirubin 0–6 μmol/L, Platelet count 101–320 × 10 (9)/L, Fibrinogen 3–5 mg/L, D-dimer < 0.55 μg/mL, Cholesterol 2.8–5.85 mmol/L, Total protein 60–80 g/L, Albumin 33–55 g/L, BNP < 100 pg/mL, Ferritin 15–200 μg/L.

**Figure 1:**
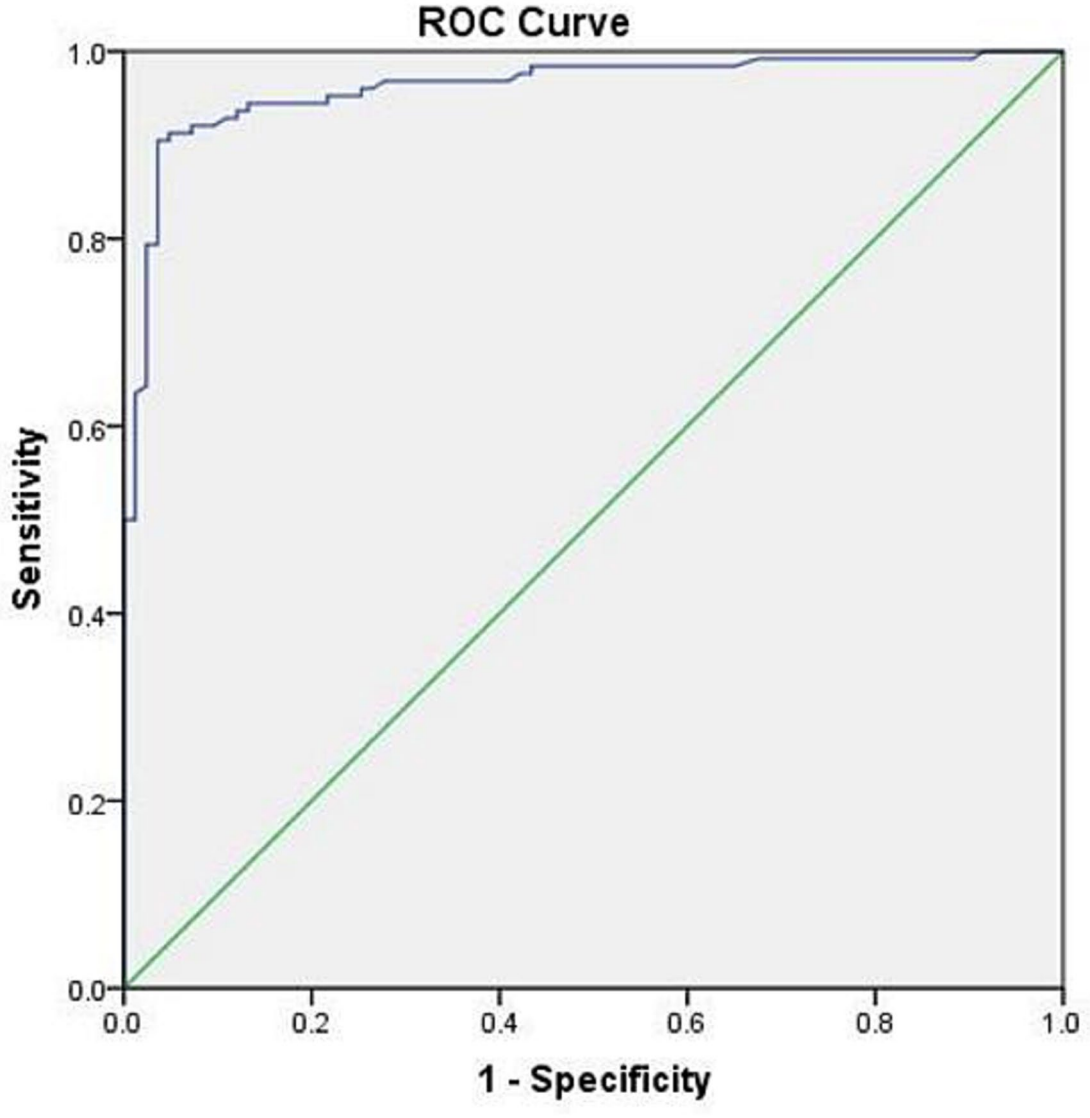
ROC curve of d-dimer (< 2 days) in postpartum women. AUC: 0.951 (95% CI 0.925–0.976, *p* < 0.001).

In this study, 125 (96.2%) postpartum women in the PE group had d-dimer levels exceeding the upper limit of the reference value (0.55 mg/L). The ROC curve analysis showed the best cutoff point for d-dimer level (< 2 days) within postpartum women was 2.24 mg/L, with a specificity of 90.1% and a sensitivity of 88.9% (Figure 1).

### Thrombosis scores and postpartum thrombosis risk assessment

**Figure 2:**
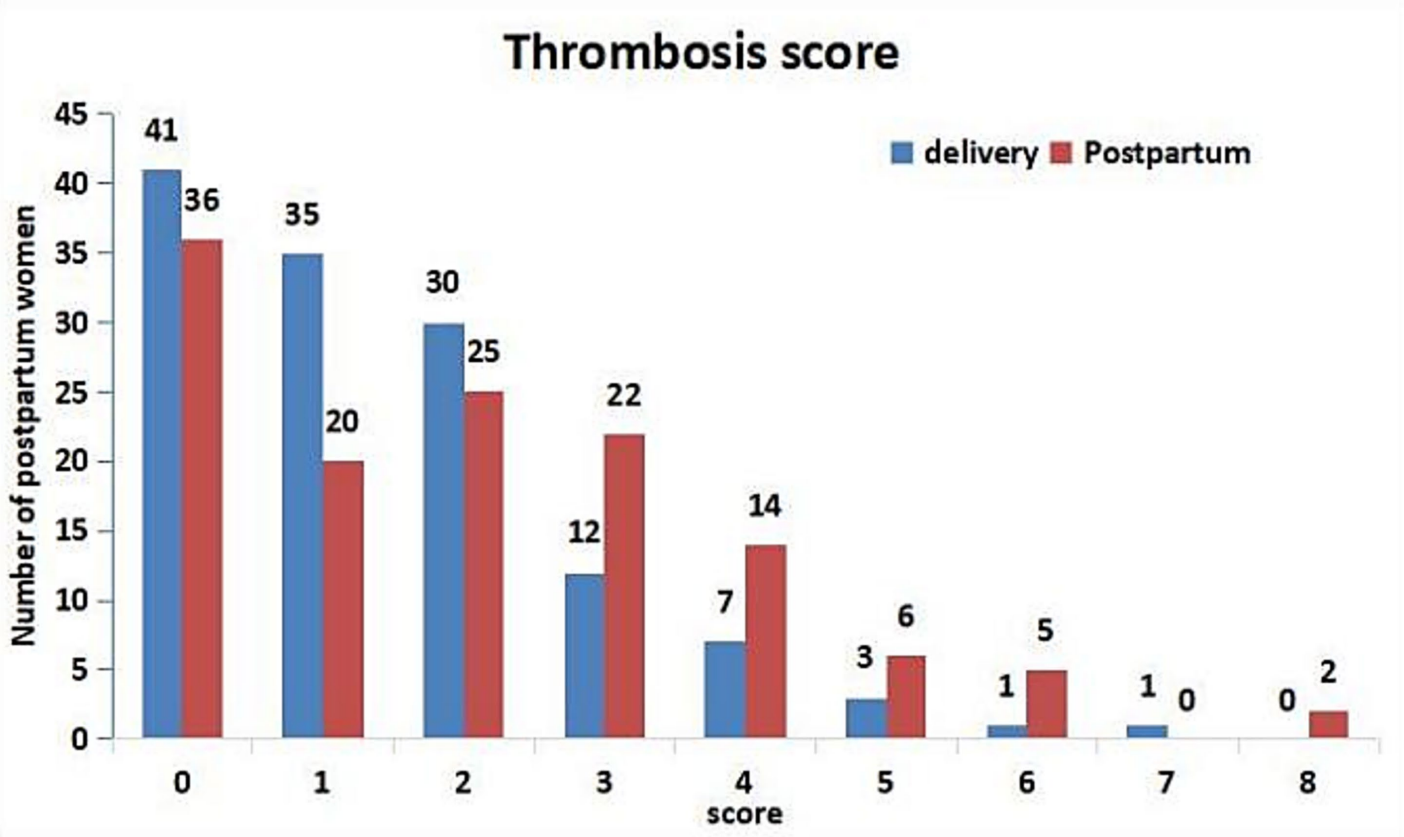
Thrombosis score of PE group on the day of delivery and postpartum. According to the Consensus on prevention and treatment of obstetric venous thromboembolism in Shanghai (7), the risk of thrombosis was divided into three grades: extremely high risk (≥ 4 points), high risk (3 points before delivery or 2–3 points after delivery), and low risk (0–1 point).

In the PE group, the thrombosis scores on the day of postpartum were significantly higher than those on the day of delivery (*p* = 0.008). The number of women with extremely high risk (≥ 4 points) and high risk (3 points postpartum) has increased significantly (7) (Figure 2). Postpartum thrombosis risk assessment: 95 persons were green (low risk), 11 persons were yellow (general risk), 5 persons were orange (higher risk), and 19 persons were red (high risk).

### Multivariate logistic regression analysis

Independent risk factors for PE were further identified by multivariable stepwise logistic regression analysis. The results revealed that VTE (OR 6.538, 95% CI 31.133–15339.681, *p* < 0.05), abnormal echocardiography (OR 4.499, 95% CI 5.636–1436.178, *p* = 0.001), and d-dimer level (OR 1.363, 95% CI 1.716–8.907, *p* = 0.001) were independent risk factors for PE (Table 4).

**Table 4:** Multivariate logistic regression analysis.

Variable	*B*	S.E.	Wald	*p*	Exp(B)	95% CI
Prophylactic anticoagulation/(%)	−0.334	1.002	0.111	0.739	0.716	0.100–5.105
Emergency c-section/(%)	0.359	1.341	0.072	0.789	1.432	0.103–19.829
Intraoperative hemorrhage/(%)	2.172	1.114	3.798	0.051	8.775	0.988–77.955
Postpartum hemorrhage/(%)	2.398	1.904	1.586	0.208	11.004	0.263–459.563
VTE/(%)	6.538	1.582	17.089	0.000	691.065	31.133–15339.681
Abnormal echocardiography/(%)	4.499	1.413	10.134	0.001	89.969	5.636–1436.178
Total bilirubin/(μmol/L)	−0.073	0.141	0.264	0.608	0.930	0.705–1.227
D-dimer/(μg/mL)	1.363	0.420	10.529	0.001	3.909	1.716–8.907
Total protein/(g/L)	0.004	0.014	0.078	0.781	1.004	0.977–1.032
Albumin/(g/L)	−0.017	0.172	0.010	0.922	0.983	0.701–1.379

### Comparison of laboratory indexes before and after anticoagulation therapy in the PE group

After the diagnosis of PE, all the parturients received anticoagulation therapy. Of these, 126 (96.9%) patients received nadroparin calcium and 4 (3.1%) patients received enoxaparin. The median number of days these patients received anticoagulation therapy was 10.0 (5.0, 14.0) days. Only one patient was switched to oral rivaroxaban after 11.0 days of nadroparin calcium. The majority of PE patients improved in clinical manifestations and laboratory indicators after anticoagulant therapy (Table 5). Seven patients improved after hospital transfer. After anticoagulant therapy, total bilirubin, fibrinogen, d-dimer, cholesterol, and BNP decreased significantly (*p* < 0.05). At the same time, urea nitrogen, ALT, platelet count, total protein, and albumin were significantly elevated (*p* < 0.05). Systolic blood pressure, diastolic blood pressure, SPO_2_, and partial pressure of oxygen did not show significant changes (*p* > 0.05). One patient had an allergic reaction; her allergic symptoms went away after she stopped taking the medicine.

**Table 5:** The results of laboratory indexes before and after anticoagulation therapy in the PE group.

Laboratory indicators^a^	PE group (before anticoagulation)	PE group (after anticoagulation)	*z*/*x*^2^	*p*
Creatinine / (μmol/L)	45.0 (40.0, 51.0)	45.0 (40.0, 51.0)	−0.458	0.647
Urea nitrogen / (mmol/L)	3.0 (2.4, 3.9)	3.6 (3.0, 4.3)	−2.838	0.005
ALT / (U/L)	13.0 (9.0, 19.5)	27.3 (13.0, 43.0)	−5.376	<0.05
AST / (U/L)	21.0 (16.0, 26.0)	23.5 (16.3, 33.8)	−1.521	0.128
Total bilirubin / (μmol/L)	8.8 (6.8, 11.2)	7.2 (6.0, 9.8)	−2.456	0.014
Direct bilirubin / (μmol/L)	1.9 (1.4, 2.6)	1.9 (1.2, 2.4)	−0.564	0.573
Platelet count / (×10^9^/L)	189.0 (158.0, 239.0)	271.0 (230.0, 315.0)	−7.657	<0.05
Fibrinogen / (mg/L)	4.0 (3.4, 4.8)	3.3 (2.7, 4.0)	−5.202	<0.05
D-dimer / (μg/mL)	5.0 (3.7, 10.5)	1.0(0.4, 1.6)	−12.004	<0.05
Cholesterol / (mmol/L)	5.8 (5.0, 6.6)	5.4 (4.6, 6.0)	−1.989	0.047
Total protein / (g/L)	53.6 (50.5, 58.0)	61.7 (55.7, 66.2)	−5.672	<0.05
Albumin / (g/L)	30.3 (28.1, 32.8)	34.9 (31.7, 39.0)	−6.081	<0.05
BNP / (pg/mL)	76.0 (38.1, 162.3)	44.5 (15.4, 78.8)	−3.872	<0.05
Ferritin / (μg/L)	25.1 (16.1, 40.1)	13.0(0.0, 25.9)	−1.135	0.300

a Creatinine 53–115 μmol/L, Urea nitrogen 2.5–6.5 mmol/L, ALT 0–40 U/L, AST 0–40 U/L, Total bilirubin 0–17.1 μmol/L, Direct bilirubin 0–6 μmol/L, Platelet count 101–320 × 10 (9)/L, Fibrinogen 3–5 mg/L, D-dimer < 0.55 μg/mL, Cholesterol 2.8–5.85 mmol/L, Total protein 60–80 g/L, Albumin 33–55 g/L, BNP < 100 pg/mL, Ferritin 15–200 μg/L.

### Subgroup analysis of emergency cesarean section or multiple pregnancy

A subgroup analysis was performed according to whether the patient underwent emergency cesarean section or multiple pregnancies to explore its effect on PE (Tables 6, 7).

**Table 6:** Subgroup analysis of emergency cesarean section.

Emergency cesarean section				
Variable	non-PE Group (*n* = 11)	PE Group (*n* = 27)	*z*/*x*^2^	*p*
Age / (years)	30.0 (28.0, 35.0)	33.0 (29.0, 34.0)	−0.569	0.590
Number of births ≥ 1 time / (%)	2.0 (18.2)	6.0 (22.2)	0.077	1.000
Interval between births 1–3 years / (%)	1.0 (9.1)	1.0 (3.7)	0.455	0.501
Twins / (%)	2.0 (18.2)	2.0 (7.4)	0.963	0.564
No activity > 3 d / (%)	0.0 (0.0)	1.0 (3.7)	0.418	1.000
Prophylactic anticoagulation / (%)	5.0 (54.5)	21.0 (71.1)	2.051	0.238
Intraoperative hemorrhage / (%)	4.0(36.4)	26.0 (96.3)	16.891	<0.05
Postpartum hemorrhage / (%)	0.0 (0.0)	8.0 (29.6)	4.128	0.077
VTE / (%)	0.0 (0.0)	12.0 (44.4)	7.145	0.008
Abnormal echocardiography / (%)	2.0 (18.2)	12.0 (44.4)	2.317	0.160
Laboratory indicators^a^				
Creatinine / (μmol/L)	47.0 (40.0, 55.0)	46.0 (42.0, 51.5)	−0.494	0.643
Urea nitrogen / (mmol/L)	3.9 (3.1, 5.0)	2.8 (2.1, 3.8)	−1.895	0.057
Total bilirubin / (μmol/L)	5.3 (4.2, 6.8)	9.0 (6.1, 12.8)	−2.495	0.011
Fibrinogen / (mg/L)	4.4 (4.0, 4.7)	4.2 (3.2, 5.0)	−0.497	0.638
D-dimer / (μg/mL)	1.5 (0.8, 1.8)	5.9 (4.0, 11.3)	−3.888	<0.05
Total protein / (g/L)	59.5 (58.4, 63.7)	53.4 (49.9, 57.9)	−2.695	0.005
Albumin / (g/L)	34.2 (33.1, 35.7)	29.9 (27.5, 32.1)	−3.306	<0.05

a Creatinine 53–115 μmol/L, Urea nitrogen 2.5–6.5 mmol/L, Total bilirubin 0–17.1 μmol/L, Fibrinogen 3–5 mg/L, D-dimer < 0.55 μg/mL, Total protein 60–80 g/L, Albumin 33–55 g/L.

**Table 7:** Subgroup analysis of multiple pregnancies.

Multiple pregnancies				
Variable	non-PE Group (*n* = 9)	PE Group (*n* = 14)	*z*/*x*^2^	*p*
Age / (years)	32.0 (30.5, 35.5)	33.0 (29.8, 34.0)	−0.508	0.643
Number of births ≥ 1 time / (%)	1.0 (11.1)	4.0 (28.6)	0.982	0.611
Interval between births 1–3 years / (%)	0.0 (0.0)	1.0 (7.1)	0.672	1.000
Emergency cesarean section / (%)	2.0 (22.2)	2.0 (14.3)	0.240	1.000
Prophylactic anticoagulation / (%)	5.0 (55.6)	8.0 (57.1)	0.006	1.000
Intraoperative hemorrhage / (%)	5.0 (55.6)	13.0 (92.9)	4.480	0.056
Postpartum hemorrhage / (%)	1.0 (11.1)	8.0 (57.1)	4.874	0.040
VTE / (%)	1.0 (11.1)	9.0 (64.3)	6.303	0.029
Abnormal echocardiography / (%)	1.0 (11.1)	8.0 (57.1)	4.874	0.040
Laboratory indicators^a^				
Creatinine / (μmol/L)	49.4 (39.5, 74.5)	46.0 (39.5, 51.5)	−0.737	0.471
Urea nitrogen / (mmol/L)	3.1 (1.8, 4.9)	3.0 (2.7, 4.4)	−0.399	0.697
Total bilirubin / (μmol/L)	5.6 (4.2, 7.8)	8.3 (6.5, 9.7)	−2.062	0.041
Fibrinogen / (mg/L)	4.6 (4.1, 5.2)	3.4 (2.7, 4.7)	−1.503	0.144
D-dimer / (μg/mL)	1.6 (0.4, 2.4)	7.2 (3.7, 15.2)	−3.340	<0.05
Total protein / (g/L)	60.5 (52.4, 64.1)	51.9 (47.7, 58.1)	−1.883	0.064
Albumin / (g/L)	33.4 (29.0, 38.1)	30.0 (25.9, 32.6)	−2.282	0.020

a Creatinine 53–115 μmol/L, Urea nitrogen 2.5–6.5 mmol/L, Total bilirubin 0–17.1 μmol/L, Fibrinogen 3–5 mg/L, D-dimer < 0.55 μg/mL, Total protein 60–80 g/L, Albumin 33–55 g/L.

In the group of women undergoing emergency cesarean section, there was more frequent intraoperative bleeding in the PE group (26.0 vs. 4.0, *p* < 0.05), and 12.0 patients (44.4%, *p* = 0.008) had a history of VTE. In these patients, TB and d-dimer were higher, and TP and ALB were lower (*p* < 0.05).

In patients with multiple pregnancies, the pulmonary embolism group showed significantly higher rates of postpartum hemorrhage (8.0 vs. 1.0, *p* = 0.040), VTE history (9.0 vs. 1.0, *p* = 0.029), and abnormal echocardiography (8.0 vs. 1.0, *p* = 0.040). These patients also exhibited elevated TB and d-dimer levels, while ALB levels were notably lower (*p* < 0.05).

## Discussion

The incidence of thromboembolic events in perinatal women was higher than that in the general population. This was due to changes in the coagulation system during pregnancy (8). A hypercoagulable state during pregnancy may protect a woman from bleeding during a miscarriage or delivery. However, there was a natural “Virchow triad” (the three elements of thrombosis) in pregnant women: blood stasis, vascular or endothelial injury, and hypercoagulability. Thus, the risk of pregnancy thrombus increased 5–10 times, and postpartum thrombus risk increased 20 times (9). Among them, pulmonary embolism was the leading cause of malignant death during the perinatal period; if not found in time or improperly managed, 20% of patients will die (1). However, the clinical symptoms of PE patients were often atypical. 87.7% of postpartum women had no clinical manifestations when PE was diagnosed, but these people may have had VTE, echocardiographic abnormalities, or other risk factors. Therefore, this study aimed to diagnose pulmonary embolism in postpartum women and analyze its possible risk factors.

According to Wang ZL et al., age, medical comorbidities, number of births, previous history of VTE, obesity, varicose veins, and assisted reproductive technology (ART) were identified as major risk factors (10). In this study, the age of the PE group (33.0 years) was significantly higher than that of the non-PE group (31.0 years) (*p* < 0.05). However, there were no significant differences between the two groups in terms of weight (childbirth), BMI, SBP, DBP, number of pregnancies, newborn weight, number of IVF patients, thrombosis-related diseases, and medical complications (*p* > 0.05). 62.3% (*n* = 81, *p* = 0.008) of the women in the PE group were primiparous, which was significantly lower than the non-PE group. 37.7% of the mothers in the PE group were parous, and the interval between the birth of two children was mostly 1–3 years; the difference between the two groups was statistically significant (*p* < 0.05).

Galambosi PJ et al. also revealed several independent risk factors in postpartum women, such as thrombophilia, gestational diabetes, premature birth, anemia, chorioamnionitis, and postpartum hemorrhage (11). Although our study revealed that there was a significant difference between the two groups in d-dimer elevated during pregnancy, anemia, prenatal fever, emergency caesarean section, twins, acute postpartum infection, intraoperative hemorrhage, and postpartum hemorrhage (p < 0.05), none of them was an independent risk factor for PE. The cognition of multiple pregnancy was not consistent. Jacobsen AF et al. demonstrated that multiple pregnancy was a risk factor for PE (12). However, Che Ronghua et al. reported that multiple pregnancy was a protective factor for the development of PE (1). Multiple pregnancies are more common in elderly pregnant women and patients undergoing assisted reproductive techniques, leading to a high pregnancy thrombus score; therefore, prophylactic anticoagulation before delivery can effectively prevent thrombus.

Blondon M et al. showed that the risk of PE was more than 4 times higher in emergency caesarean section (13). Similar results were obtained from our country’s large sample data (10). In our study, emergency caesarean sections were also more common in the PE group (20.8% vs. 5.7%, *p* < 0.05). In addition, it was found that 6.9% of acute postpartum infections occurred in the PE group (*n* = 9.0, *p* < 0.05); six postpartum women may be related to an emergency caesarean section. Jacobsen AF et al. found that termination of pregnancy, stillbirth, intrauterine growth retardation (IUGR), pre-eclampsia, and prenatal hemorrhage were also risk factors for PE (12). There were no similar results found in our study.

The high-risk factors of venous thrombosis were also the high-risk factors of PE, so patients with VTE were more likely to develop PE (1). Current research found that about one-third of patients with VTE manifestations also had PE (14). Within 48 h after the delivery of the placenta, postpartum hemorrhage caused by non-endogenous coagulation system factors could promote increased fibrinogen release; the endogenous coagulation pathway was relatively hyperactive, and the damage of the venous wall could further promote a decrease in activated partial thromboplastin time, increasing the incidence of venous thrombosis. With the detachment of venous thrombi, they can block the pulmonary vessels, leading to the occurrence of PE. It is generally believed that most PEs are complications of VTE (1). VTE was the main risk factor for the occurrence of PE in pregnant women during pregnancy and the puerperium (10). Our research had found similar results; the VTE was an independent risk factor for PE (OR 6.538, 95% CI 31.133–15339.681, *p* < 0.05), in postpartum women with VTE, being 6.538 times higher than that in normal postpartum women. Therefore, it was necessary to monitor the possibility of PE in patients with VTE manifestations in clinical practice.

JK et al. pointed out that echocardiography was the most commonly used method for the diagnosis and treatment of acute pulmonary embolism; it also provides clues of hemodynamic instability in emergency situations (15). Nasser MF et al. found that the results of echocardiography examination have prognostic value for the treatment of pulmonary embolism (16). Similar statements were also found in the guidelines of the European Society of Cardiology and the American Heart Association (17, 18), mainly manifested as right ventricular dysfunction. Our research also had similar findings: 42.3% (*n* = 55.0, *p* < 0.05) of people with abnormal echocardiographic changes in the PE group. Among them, 10.9% (*n* = 6) of people had right ventricular dilatation. Echocardiographic abnormalities were also an independent risk factor (OR 4.499, 95% CI 5.636–1436.178, *p* = 0.001) for PE in postpartum women. Although, there was no statistically significant difference between the two groups among pulmonary artery hypertension (*n* = 3.0, 2.3%), left atrial enlargement (*n* = 3.0, 2.3%), artery widening (*n* = 1.0, 0.8%), pericardial effusion (*n* = 4.0, 3.1%), and left ventricular diastolic (*n* = 4.0, 3.1%). We should attach importance to the value of echocardiography in the diagnosis of PE in parturients. More importantly, echocardiography had no known adverse effects on pregnant women and fetuses, especially for those postpartum women who were suspected of PE but had atypical clinical manifestations and inconclusive routine laboratory tests.

The level of creatinine (45.0 μmol/L), urea nitrogen (3.0 mmol/L), total protein (53.6 g/L), and albumin (30.3 g/L) in the PE group was lower (*p* < 0.05), and the level of total bilirubin (8.8 μmol/L) was higher (p < 0.05). These changes in creatinine, urea nitrogen, and total bilirubin were related to the worse nutritional status of women in the PE group. Malnutrition reduces the production of urea nitrogen, and a decrease in albumin leads to a decrease in indirect bilirubin entry into the liver, which in turn leads to an increase in total bilirubin levels. Therefore, the nutritional status of postpartum women is important. The levels of total protein and albumin in the PE group were lower than the normal range, which indicated that the nutritional status of postpartum women was poor. The poorer the nutritional status of postpartum women, the more likely they are to send PE.

Elevated fibrinogen can lead to hypercoagulability, which increases the risk of thrombosis. However, in the early stages of thrombosis, large amounts of thrombin (IIA) activate fibrinogen rapidly and convert it to fibrin. In this study, we monitored fibrinogen levels at the time of PE diagnosis. We found that fibrinogen levels were significantly lower in the PE group than in the non-PE Group (*p* < 0.05). Similar results were observed in studies by Hu et al. (19) but neither they nor we found fibrinogen to be an independent risk factor for PE.

D-dimer is often used as a marker of recent thrombosis in clinical study (20). It can be used as a preliminary screening tool for pulmonary embolism, offering the advantages of economy, rapidity, and high sensitivity (21). However, the level of d-dimer in pregnancy was higher than that in non-pregnancy and increased with the increase of gestational age and decreased gradually after delivery. Domestic and foreign studies have shown that the concentration of d-dimer in the blood of postpartum women drops to the pre-labor level 48 h after delivery, but it was still high (22, 23). Furthermore, Réger et al. (24) pointed out that most healthy perinatal women also had d-dimer values above the normal reference range during pregnancy and the puerperium. Therefore, studies have demonstrated the predictive value of d-dimer detection for pregnancy-related thrombosis by raising the cut-off or looking for a higher reference range for d-dimer (22). The role of d-dimer in the screening of PE in perinatal women is controversial. Grossman et al. noted that routine d-dimer thresholds (500 μg/L) are almost meaningless when acute pulmonary embolism is excluded during the perinatal period (25). Domestic and foreign guidelines do not recommend the use of d-dimer in PE screening and diagnosis in perinatal women (26, 27). However, there was a significant difference in d-dimer between the two groups at diagnosis of PE (*p* < 0.05). High d-dimer levels (OR 1.363, 95% CI 1.716–8.907, *p* = 0.001) were shown to be a specific marker for clinical monitoring of PE in multivariate analysis. Thus, d-dimer detection was necessary for postpartum women. In our study, ROC curve analysis provided us with a higher d-dimer threshold (2.24 mg/L) with higher specificity (90.1%) and sensitivity (88.9%). Higher specificity ensures a lower false-positive rate. In postpartum patients, notably elevated d-dimer levels (> 2.24 mg/L) serve as a strong warning sign, indicating a potential pulmonary embolism (PE), which should prompt clinicians to implement proactive diagnostic testing and continuous monitoring for affected patients. Higher sensitivity means only a small number of postpartum women diagnosed with PE may have d-dimer levels that do not exceed this threshold. We should pay more attention to the changes in high-risk factors and clinical manifestations in these patients, and imaging examinations can be performed when necessary. Although the current data were not sufficient to directly recommend a single d-dimer level as the sole criterion for initiating prophylactic anticoagulation therapy, the d-dimer threshold (2.24 mg/L) identified in this study was much higher than the exclusion cutoff value (0.5 mg/L) commonly used for non-pregnant populations, suggesting that the threshold may be more clinically relevant in postpartum populations. Therefore, we recommend that patients with risk factors (such as advanced age, multiple childbirth history, emergency cesarean sections, twin pregnancies, postpartum acute infections, intraoperative or postpartum hemorrhage, VTE history, and abnormal echocardiograms, etc.) should undergo d-dimer monitoring. Clinical manifestations and test results should be combined to assess risk levels, and clinical guidelines for prophylactic or therapeutic anticoagulation should be strictly followed (4, 6).

BNP is a sensitive marker of ventricular dysfunction. When PE causes an increase in pulmonary vascular resistance or pulmonary hypertension, it can lead to an increase in ventricular wall tension and increase the synthesis and secretion of BNP. BNP levels tend to rise earlier than other clinical symptoms, so that the BNP level can be used as an early indicator of the risk and extent of PE and right ventricular dysfunction. Tanabe Y et al. found that BNP was associated with the risk and severity of acute PE (28). As PE progressed from low risk to high risk, the BNP level increased significantly, which was an index for screening PE and evaluating the prognosis of patients with acute PE. This study also found that the level of BNP in the PE group was significantly higher than that in the non-PE group (*p* < 0.05). Although BNP was not an independent risk factor for PE, the elevation of BNP levels may also be a warning sign of the occurrence of PE in those with obvious clinical symptoms or atypical symptoms, which should be paid attention to.

According to the systematic review, maternal and infant survival rates were higher (94% vs. 88%) in high-risk PE women treated with anticoagulation (29). In the present study, all parturients with high-risk factors and clinical manifestations were given appropriate prophylactic anticoagulation, and all patients with a definite diagnosis were given appropriate therapeutic anticoagulation. All patients received anticoagulant therapy in accordance with the guidelines and showed significant improvement in clinical symptoms and laboratory indicators after anticoagulant treatment (*p* < 0.05), with high maternal (100.0%) and infant (98.5%) survival rates. Although no bleeding occurred in our study, the use of anticoagulation therapy in clinical cases of PE requires a balance between thrombosis and bleeding risks, with more individualized treatment and monitoring when necessary.

In subgroup analysis based on emergency cesarean status, intraoperative bleeding was more common (*p* < 0.05), while in subgroup analysis based on multiple pregnancies, postpartum bleeding was more common (*p* < 0.05). The difference between the two subgroup analyses was related to the mode of delivery and injury during delivery. In the above two subgroup analyses, we observed significant differences between groups in VTE history, TB levels, d-dimer levels, and ALB levels (*p* < 0.05). However, none of these risk factors remained independent predictors of PE (*p* > 0.05), which may be attributed to the relatively small sample size in these subgroup analyses.

## Conclusion

In this study, we found that venous thromboembolism and abnormal echocardiography were the independent risk factors of PE. Therefore, particular attention should be given to those with high-risk factors, such as VTE, abnormal ECGs, or clinical symptoms related to PE. For these postpartum women, d-dimer levels should be monitored, and a comprehensive assessment should be made. A significantly elevated d-dimer level (> 2.24 mg/L) serves as strong evidence of potential PE development, prompting clinicians to prioritize imaging studies for early detection and confirmation. Although it is not recommended to use elevated d-dimer (> 2.24 mg/L) alone for the screening and diagnosis of pulmonary embolism in postpartum women, this indicator may indicate that the patient needs appropriate prophylactic and therapeutic anticoagulation, which can improve the prognosis, especially in postpartum women with confirmed pulmonary embolism.

## Limitations

Although we included a significant number of PE patients (*n* = 130), the overall sample size was still relatively limited. This may result in our study lacking sufficient statistical power to detect statistically significant associations between some smaller factors and PE. The sample size limits our ability to perform more in-depth subgroup analysis and the universality of the results. To overcome this limitation, we will subsequently establish a large, multicenter cohort study to systematically collect clinical data and outcomes from postpartum women over a longer period of time, providing a more solid clinical basis for the risk assessment of postpartum PE.

## Data Availability

The raw data supporting the conclusions of this article will be made available by the corresponding author on reasonable request.

## References

[1] Che R Pei J Wan S Li H Hua X. Analysis of clinical high risk factors and biological indexes in parturients with pulmonary embolism. J Tongji Univ. (2022) 43:63–9. doi: 10.12289/j.issn.1008-0392.21152

[2] Liu S Rouleau J Joseph KS Sauve R Liston RM Young D. Epidemiology of pregnancy-associated venous thromboembolism: a population-based study in Canada. J Obstet Gynaecol Can. (2009) 31:611–20. doi: 10.1016/S1701-2163(16)34240-2, PMID: 19761634

[3] Lucassen W Geersing GJ Erkens PM Reitsma JB Moons KG Büller H. Clinical decision rules for excluding pulmonary embolism: a meta-analysis. Ann Intern Med. (2011) 155:448–60. doi: 10.7326/0003-4819-155-7-201110040-00007, PMID: 21969343

[4] Chinese Society of Cardiolog. Chinese expert consensus on the treatment of acute pulmonary embolism by the multidisciplinary team. Zhonghua Xin Xue guan Bing Za Zhi. (2022) 50:25–35. doi: 10.3760/cma.j.cn112148-20210527-00455 35045611

[5] Tromeur C van der Pol LM Le Roux PY Ende-Verhaar Y Salaun PY Leroyer C. Computed tomography pulmonary angiography versus ventilation-perfusion lung scanning for diagnosing pulmonary embolism during pregnancy: a systematic review and meta-analysis. Haematologica. (2019) 104:176–88. doi: 10.3324/haematol.2018.19612, PMID: 30115658 PMC6312023

[6] Group of pulmonary embolism and pulmonary vascular disease, respiratory branch of Chinese Medical Association. Guidelines for the diagnosis, treatment and prevention of pulmonary thromboembolism. Chin J Med. (2018) 98:1060–87. doi: 10.3760/cma.j.issn.0376-2491.2018.14.007

[7] Li X Di W Gu H Tao M Cheng W Ying H. Consensus on prevention and treatment of obstet ricvenous thromboembolism in Shanghai. Shanghai Med J. (2020) 43:645–50. doi: 10.19842/j.cnki.issn.0253-9934.2020.11.001

[8] Kane EV Calderwood C Dobbie R Morris C Roman E Greer IA. A population-based study of venous thrombosis in pregnancy in Scotland 1980-2005. Eur J Obstet Gynecol Reprod Biol. (2013) 169:223–9. doi: 10.1016/j.ejogrb.2013.03.024, PMID: 23684606

[9] Kalaitzopoulos DR Panagopoulos A Samant S Ghalib N Kadillari J Daniilidis A. Management of venous thromboembolism in pregnancy. Thromb Res. (2022) 211:106–13. doi: 10.1016/j.thromres.2022.02.002, PMID: 35149395

[10] Wang ZL Geng HZ Zhao XL Zhu QY Lin JH Zou L. Survey of related factors of maternal venous thromboembolism in nine hospitals of China. Zhonghua Fu Chan Ke Za Zhi. (2020) 55:667–72. doi: 10.3760/cma.j.cn112141-20200414-00326, PMID: 33120477

[11] Galambosi PJ Gissler M Kaaja RJ Ulander VM. Incidence and risk factors of venous thromboembolism during postpartum period: a population-based cohort-study. Acta Obstet Gynecol Scand. (2017) 96:852–61. doi: 10.1111/aogs.13137, PMID: 28369660

[12] Jacobsen AF Skjeldestad FE Sandset PM. Incidence and risk patterns of venous thromboembolism in pregnancy and puerperium--a register-based case-control study. Am J Obstet Gynecol. (2008) 198:233.e1–233.e2337. doi: 10.1016/j.ajog.2007.08.041 17997389

[13] Blondon M Casini A Hoppe KK Boehlen F Righini M Smith NL. Risks of venous thromboembolism after cesarean sections: a Meta-analysis. Chest. (2016) 150:572–96. doi: 10.1016/j.chest.2016.05.021, PMID: 27262227

[14] White RH. The epidemiology of venous thromboembolism. Circulation. (2003) 107:I4–8. doi: 10.1161/01.CIR.0000078468.11849.66 12814979

[15] Oh JK Park JH. Role of echocardiography in acute pulmonary embolism. Korean J Intern Med. (2023) 38:456–70. doi: 10.3904/kjim.2022.273, PMID: 36587934 PMC10338244

[16] Nasser MF Jabri A Limaye S Sharma S Hamade H Mhanna M. Echocardiographic evaluation of pulmonary embolism: a review. J Am Soc Echocardiogr. (2023) 36:906–12. doi: 10.1016/j.echo.2023.05.006, PMID: 37209948

[17] Konstantinides SV Meyer G Becattini C Bueno H Geersing GJ Harjola VP. 2019 ESC guidelines for the diagnosis and management of acute pulmonary embolism developed in collaboration with the European Respiratory Society (ERS). Eur Heart J. (2020) 41:543–603. doi: 10.1093/eurheartj/ehz405, PMID: 31504429

[18] Jaff MR McMurtry MS Archer SL Cushman M Goldenberg N Goldhaber SZ. Management of massive and submassive pulmonary embolism, iliofemoral deep vein thrombosis, and chronic thromboembolic pulmonary hypertension: a scientific statement from the American Heart Association. Circulation. (2011) 123:1788–830. doi: 10.1161/CIR.0b013e318214914f, PMID: 21422387

[19] Hu W Xu D Li J Chen C Chen Y Xi F. The predictive value of D-dimer test for venous thromboembolism during puerperium in women age 35 or older: a prospective cohort study. Thromb J. (2020) 18:26. doi: 10.1186/s12959-020-00241-y, PMID: 33088222 PMC7566136

[20] Parilla BV Fournogerakis R Archer A Sulo S Laurent L Lee P. Diagnosing pulmonary embolism in pregnancy: are biomarkers and clinical predictive models useful? AJP Rep. (2016) 6:e160–4. doi: 10.1055/s-0036-1582136, PMID: 27119048 PMC4844549

[21] Xi X Yang J Wang Z Zhu C Li J Liu S. The value of adjusting d-dimer threshold according to renal function in the diagnosis of pulmonary embolism. Chin J Med. (2015) 95:2433–6. doi: 10.3760/cma.j.issn.0376-2491.2015.30.006 26711203

[22] Xu D Cai SP Xu JW Liang C He J. Study on the dynamic changes of D-dimer during pregnancy and early puerperium. Zhonghua Fu Chan Ke Za Zhi. (2016) 51:666–71. doi: 10.3760/cma.j.issn.0529-567X.2016.09.006, PMID: 27671047

[23] Murphy N Broadhurst DI Khashan AS Gilligan O Kenny LC O’Donoghue K. Gestation-specific D-dimer reference ranges: a cross-sectional study. BJOG. (2015) 122:395–400. doi: 10.1111/1471-0528.12855, PMID: 24828148

[24] Réger B Péterfalvi A Litter I Pótó L Mózes R Tóth O. Challenges in the evaluation of D-dimer and fibrinogen levels in pregnant women. Thromb Res. (2013) 131:e183–7. doi: 10.1016/j.thromres.2013.02.005, PMID: 23481480

[25] Grossman KB Arya R Peixoto AB Akolekar R Staboulidou I Nicolaides KH. Maternal and pregnancy characteristics affect plasma fibrin monomer complexes and D-dimer reference ranges for venous thromboembolism in pregnancy. Am J Obstet Gynecol. (2016) 215:466.e1–8. doi: 10.1016/j.ajog.2016.05.013, PMID: 27179442

[26] Kakkos SK Gohel M Baekgaard N Bauersachs R Bellmunt-Montoya S Black SA. Editor’s choice - European Society for Vascular Surgery (ESVS) 2021 clinical practice Guidelines on the Management of Venous Thrombosis. Eur J Vasc Endovasc Surg. (2021) 61:9–82. doi: 10.1016/j.ejvs.2020.09.023, PMID: 33334670

[27] Department of Obstetrics and Gynecology, Chinese Medical Association. Experts consensus on prevention and treatment of venous thromboembolism during pregnancy and puerperium. Chin J Obstet Gynecol. (2021) 56:236–43. doi: 10.3760/cma.j.cn112141-20201110-00826

[28] Tanabe Y Obayashi T Yamamoto T Takayama M Nagao K. Predictive value of biomarkers for the prognosis of acute pulmonary embolism in Japanese patients: results of the Tokyo CCU network registry. J Cardiol. (2015) 66:460–5. doi: 10.1016/j.jjcc.2015.03.002, PMID: 25843673

[29] Martillotti G Boehlen F Robert-Ebadi H Jastrow N Righini M Blondon M. Treatment options for severe pulmonary embolism during pregnancy and the postpartum period: a systematic review. J Thromb Haemost. (2017) 15:1942–50. doi: 10.1111/jth.13802, PMID: 28805341

